# Biologically Active Compounds from Goji (*Lycium Barbarum* L.) Leaves Aqueous Extracts: Purification and Concentration by Membrane Processes

**DOI:** 10.3390/biom10060935

**Published:** 2020-06-21

**Authors:** Carmela Conidi, Enrico Drioli, Alfredo Cassano

**Affiliations:** 1 Institute on Membrane Technology, ITM-CNR, Via P. Bucci 17/C, 87036 CS Rende, Italy; e.drioli@itm.cnr.it; 2 Department of Engineering and of the Environment, University of Calabria, Via P. Bucci 45/A, 87036 CS Rende, Italy; 3 State Key Laboratory of Materials-Oriented Chemical Engineering, College of Chemical Engineering, Nanjing Tech University, Nanjing 210009, China; 4 Center of Excellence in Desalination Technology, King Abdulaziz University (KAU-CEDT), Jeddah 21589, Saudi Arabia

**Keywords:** Goji leaves, aqueous extraction, membrane processes, antioxidants, bioactive compounds

## Abstract

Goji (*Lycium barbarum* L.) leaves and fruits have been described as a valuable source of bioactive compounds with a great potential for the development of health-promoting formulations. The present study aimed to evaluate the potential of a sustainable process for the recovery of phenolic compounds from Goji leaves through a combination of aqueous extraction and membrane-based operations. Water was used as a safe, cheap, and non-hazardous extraction solvent, and parameters of extraction of dried Goji leaves were optimized in order to maximize the yield of polyphenols, total soluble solids (TSS), and total antioxidants simultaneously. The aqueous extract was clarified by ultrafiltration and then processed with three flat-sheet polyethersulphone (PES) membranes with molecular weight cut-off (MWCO) values in the range of 0.3–4.0 kDa, in order to remove sugar compounds from polyphenols and improve the antioxidant activity of the produced fractions. Among the selected membranes, a 1 kDa membrane exhibited the best performance in terms of purification of polyphenols from the clarified aqueous extract. The rejection by this membrane of TSS and total carbohydrates was in the range of 15.8–25.3%, and was decreased by increasing the volume reduction factor (VRF). On the other hand, the retention values for total polyphenols and total antioxidant activity (TAA) were in the range of 73–80%, and were increased by increasing the VRF.

## 1. Introduction

*Lycium barbarum* L., commonly known as Goji, has long been used in traditional Chinese medicine, and is increasingly becoming popular as a so-called “superfruit” in Europe and North America [1]. Extracts from *L. barbarum* fruit have been shown to possess a range of biological activities, including effects on aging, neuroprotection, anti-fatigue/pro-endurance, increased metabolism, glucose control in diabetics, glaucoma, antioxidant properties, immunomodulation, anti-tumor activity, and cytoprotection [2]. Therefore, Goji fruits have been widely used recently as concentrated extracts and as functional ingredients for designing innovative functional products such as juice, cake, soup, snacks, yoghurt, medicinal foods, cosmetics, and cosmeceutics [3].

On the other hand, few studies have been published until now on the leaves of the Goji plant despite their pharmacological and nutraceutical properties. They have been used as tea, medicinal vegetables, and herbal drugs in China and Southeast Asia, and are nowadays highly emphasized in Europe and North America as a functional tea or in dietary supplements [4,5]. Flavonoids have been reported as the main functional components in *L. barbarum* leaves [6]. These compounds have great potential in blocking the production of the messaging molecules that promote inflammation phenomena and protecting low-density lipoprotein (LDL) cholesterol from oxidative stress, which has been shown to reduce the onset of atherosclerosis.

Dong et al. [7], identified rutin as the predominant flavonoid of Goji leaves. This compound has been recognized for its anti-UV capacity; therefore, cultivated leaves might be good sources for anti-radiation food or anti-UV cosmetics. Other polyphenols including quercetin, isoquercitrin, chlorogenic acid, cryptochlorogenic acid, isochlorogenic acid, p-coumaric acid, luteolin, kaempferol, and caffeic acid, have been found in the leaves of Goji berries [8]. All these compounds are of great interest in treating a wide variety of diseases.

In addition, Goji leaves have been described as a sustainable source of antioxidant compounds [9,10]. The biological properties have been related to complementary, additive, or synergistic interactions between the high content of vitamins, minerals (mainly calcium, iron, and zinc), and a diversity of polyphenols, alkaloids, and polysaccharides [11,12]. Among these polyphenols are gaining more and more interest for their integration into nutraceuticals, functional foods, and cosmetics [13].

The development of an efficient methodology for the extraction, recovery, and purification of phenolic compounds from Goji leaves is a crucial step for designing new high-added-value formulations that can potentially be used as ingredients by the pharmaceutical and food sectors, and therefore increase the use of natural side-streams as raw materials to obtain bioactive-rich extracts.

Conventional methods to recover polyphenols from plant materials are based on the use of maceration assisted by organic solvents such as methanol, hexane, etc. However, long extraction times, environmental toxicity, consumption of large quantities of organic solvents, and safety aspects linked to the handling of these substances are the major drawbacks arising from these methods [14,15].

Non-conventional extraction techniques, such as pressurized liquid extraction, ultrasonic-assisted extraction (UAE), and microwave-assisted extraction (MAE), have been also applied and developed [12,16]. However, these techniques still require organic solvents or complex operations; in addition, they are characterized by partial oxidation and degradation of the compounds of interest, low extraction efficiency and selectivity, and high cost of some equipment at the industrial level. Thus, there is an increasing interest in developing green extraction technologies that are safe, fast, and easy to implement, in order to maximize polyphenol recovery while maintaining their chemical integrity and, consequently, their functional activities.

The challenge here is the development of suitable downstream processing techniques, allowing for the recovery of these compounds from their original sources without affecting their structure and function, which ultimately translates into their bioactivity. In this context, membrane processes offer interesting sustainable solutions to this problem, since they can operate in mild operating conditions of temperature and pressure, without the use of chemical agents or solvents, thus avoiding product contamination and preserving the biological activity of target compounds [17,18]. The large variety of membrane materials available, as well as the diversity of membrane processes developed, offers interesting perspectives for selecting membranes and membrane processes for specific tasks. Additional advantages include high selectivity, easy scale-up, low energy consumption, and low capital and labor costs [19,20]. In particular, pressure-driven membrane processes, such as ultrafiltration (UF) and nanofiltration (NF) have been successfully used in the fractionation and concentration of biologically active compounds, including polyphenols, from undervalued bioresources and natural products [21,22]. These processes are based on the principle of selective permeation of molecules through semipermeable membranes under a mechanical pressure as a driving force. The separation mechanism in both UF and NF processes is mainly based on a sieving effect and particles are separated according to their dimensions, although other factors, such as shape and charge, as well as interactions between the membrane itself and particles being filtered, play key roles in the separation mechanism. In particular, most UF membranes have molecular weight cut-off (MWCO) values between 1000 and 100,000 Da and pore sizes between 2 and 100 nm. NF membranes are characterized by narrow pore sizes from 0.5 to 2 nm, retaining micromolecules with molecular weights from 150 to 1000 Da [23].

To the best of our knowledge, no scientific references to date deal with the recovery of bioactive compounds from *L. barbarum* extracts via membrane processes. The present work focused on the recovery of phenolic compounds from Goji leaves through a combination of aqueous extraction and membrane operations. Specifically, in the first part of the work, parameters of extraction of dried Goji leaves, using water as safe, cheap, and non-hazardous extraction solvent, were optimized in order to maximize the yield of polyphenols, total soluble solids (TSS), and total antioxidants simultaneously. In the second part of the experimental work, the extract was clarified by UF and then processed with three flat-sheet membranes in polyethersulphone, with different MWCO values, in order to remove sugar compounds from polyphenols and improve the antioxidant activity of the produced fractions. The performance of selected membranes was compared in terms of productivity (permeate fluxes), fouling index, cleaning efficiency, and selectivity towards target compounds.

## 2. Materials and Methods

### 2.1. Materials and Chemicals

Goji leaves were provided by Favella (Corigliano Calabro, Cosenza, Italy). The fresh leaves were washed to remove impurities such as dust and then dried at 40 °C for 48 h until they reached constant weight. Dried leaves were ground into a fine powder in order to increase the contact area between extracting solvent and the solid material, and then stored in plastic containers at room temperature until use. Distilled water was used as solvent for the extraction process. Folin–Ciocalteu phenol reagent, gallic acid, potassium persulfate, 2,2′-azinobis (3-ethylbenzothiazoline-6-sulfonic acid) diammonium salt (ABTS), 6-hydroxy-2,5,7,8-tetramethylchroman-2-carboxylic acid (Trolox), sulfuric acid, glucose, and phenol were purchased from Sigma Aldrich (Milan, Italy). Sodium chloride, sodium carbonate, sodium hydrogen, and dihydrogen phosphate were purchased from Carlo Erba (Milan, Italy). All chemicals used in the experiments were of analytical grade.

### 2.2. Aqueous Extraction and Pre-Treatment

The aqueous extraction of antioxidant compounds from Goji leaves was optimized in terms of liquid-to-solid ratio (L/S) (from 4 to 20 mL/g), extraction temperature (from 40 to 90 °C), and pH (from 2 to 12) at a selected extraction time of 30 min. The aqueous extract produced under optimized operating conditions was stored in the freezer (−20 °C) until usage.

The aqueous extract was ultrafiltered using a laboratory pilot unit equipped with a capillary polysulphone (PS) membrane supplied by China Blue Star Membrane Technology Co., Ltd. (Beijing, China) having a nominal molecular weight cut-off (MWCO) of 100 kDa and an effective membrane area of 0.16 m^2^. The UF system was operated at a temperature (T) of 26 ± 1 °C and a transmembrane pressure (TMP) of 0.75 bar according to the batch concentration mode (collecting separately the permeate stream and recycling the retentate stream in the feed reservoir) until a volume reduction factor (VRF) of 6 was reached. The clarified solution was then stored at −18 °C until usage in the fractionation step. After the clarification step, the UF membrane was first washed with distilled water (at 40 °C for 30 min) and then cleaned with an enzymatic detergent (Ultrasil 50, Henkel Chemicals Ltd., Dusseldorf, Germany) (1%, 40 °C, 60 min). A final rinsing with distilled water for 20 min was then applied.

### 2.3. Fractionation of the Clarified Extract

The clarified aqueous extract was fractionated using three polyethersulphone (PES) flat-sheet membranes (from Microdyn-Nadir, Wiesbaden, Germany) with molecular weight cut-offs (MWCOs) in the range of 0.3–4.0 kDa and effective surface areas of 35.24 cm^2^. The properties of the selected membranes are reported in Table 1.

Cross-flow UF and NF experiments were performed using a laboratory bench plant supplied by Three-Es Srl (Milano, Italy) and equipped with a stainless steel circular cross-flow cell. The plant consisted of a feed tank with a capacity of 2 L, a positive displacement high pressure pump, two manometers for measuring the inlet and outlet pressures, a digital flowmeter, a pressure control valve on the retentate side, and a cooling coil fed with tap water to control the feed temperature. The permeate flux was periodically monitored by measurement of its weight, using an electronic balance (with an accuracy of 0.1 g) placed under the permeate vessel.

Experiments were conducted in total recirculation mode (both permeate and retentate were recycled to the feed tank) in order to study the effect of TMP on the permeate flux and selectivity towards bioactive compounds. The TMP value was varied in the range of 4–20 bar at a constant feed flowrate (530 L/h) and an operating temperature of 26 ± 1 °C.

For each membrane, the permeate flux was measured by increasing the TMP value from the lowest to the highest one. Once a steady-state flux was reached at a given TMP, the pressure was increased to the next value. Samples of feed, retentate, and permeate were collected for each investigated TMP and stored at −20 °C until analysis. Approximately 1.5 L of clarified aqueous extract was used for each experiment.

The 1 kDa membrane experiments were also performed in batch concentration configuration at selected operating conditions of temperature (26 ± 1 °C), TMP (8 bar), and flowrate (585 L/h).

The permeate flux (J_p_) was determined by weighing the amount of permeate in a given time through the membrane surface area according to the following equation.

(1) $$J_{p}=\frac{W_{p}}{t\cdot A}$$

where *J_p_* is the permeate flux (kg/m^2^h), *W_p_* is the weight of permeate (kg) at time *t* (h), and *A* is the membrane surface area (m^2^).

The retention of UF and NF membranes towards compounds of interest, depending on whether evaluated in total recycle or batch concentration mode, was calculated according to the following equations, respectively.

(2) $$R=\left(1-\frac{C_{p}}{C_{f}}\right)\cdot 100$$

(3) $$R=\left(1-\frac{C_{p}}{C_{r}}\right)\cdot 100$$

where *C_p_*, *C_f_*, and *C_r_* are the concentration of specific compounds in the permeate, feed, and retentate streams, respectively.

Before treatment of the aqueous extract, each membrane was conditioned with distilled water for 1 h at 20 bar, and then the water permeability (*WP*_0_) was measured. This value was determined using the slope of the straight line obtained plotting the water flux values, measured in fixed conditions of temperature (25 °C) as function of the TMP. After the treatment with the clarified aqueous extract, membranes were rinsed with tap water for 20 min and their water permeability was measured, and then the membranes were cleaned by using a 1% Ultrasil 50 solution, at 40 °C for 60 min. At the end of the enzymatic treatment, the pure water permeability was measured again.

The fouling index (FI), expressed as a percentage drop in the water permeability, was estimated by measuring the water permeability before and after the treatment of clarified aqueous extract, according to the following equation.

(4) $$FI=\left(1-\frac{WP_{1}}{WP_{0}}\right)\cdot 100$$

where *WP*_1_ is the water permeability after the treatment of the clarified extract.

The cleaning efficiency (CE) was evaluated by using the water flux recovery method, according to the following equation.

(5) $$CE=\left(\frac{WP_{2}}{WP_{0}}\right)\cdot 100$$

where *WP*_2_ is the water permeability measured after the enzymatic cleaning.

**Figure 1:**
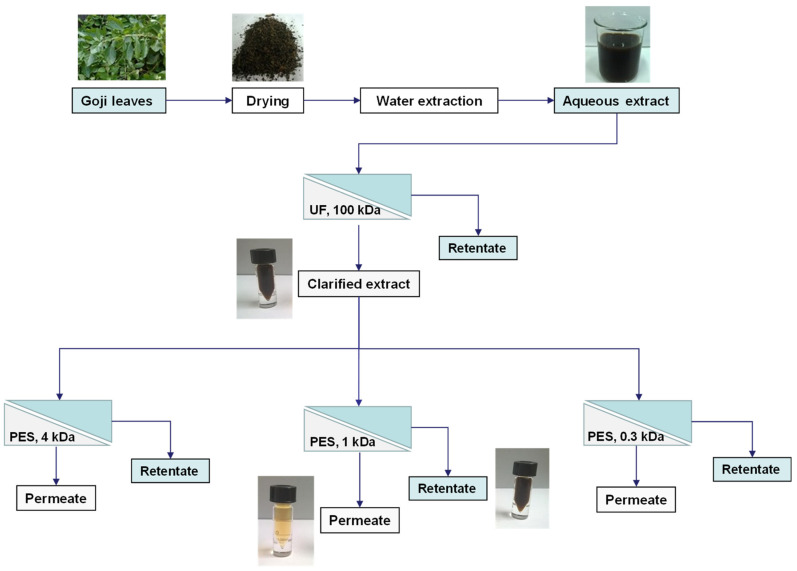
Schematic representation of the experimental design (PES, polyethersulphone).

A schematic representation of the investigated process is reported in Figure 1.

### 2.4. Measurement of Total Soluble Solids, Total Suspended Solids, and pH

Total soluble solids (TSS), expressed as °Brix, were estimated using a hand refractometer (Atago Co., Ltd., Tokyo, Japan) with scale range of 0–32 °Brix. Measurements were carried out at 20 °C. Total suspended solids were determined by centrifuging 15 mL of a pre-weight sample at 4000 rpm for 20 min; the weight of settled solids was determined after removing the supernatant. The pH of Goji leaf aqueous extracts was measured using an Orion Expandable ion analyzer EA 920 pH meter (Allometrics Inc., Webster, USA) with automatic temperature compensation.

### 2.5. Total Carbohydrates

Total carbohydrates were measured using the phenol–sulfuric acid method [27]. A sample aliquot (0.2 mL) of a carbohydrate solution was mixed with 1 mL of 5% aqueous solution of phenol in a test tube. Next, 5 mL of concentrated sulfuric acid was added rapidly to the mixture. After allowing the test tubes to stand for 10 min, they were vortexed for 30 s and placed for 30 min in a water bath at room temperature for color development. The absorbance was measured at 420 nm using a UV–visible spectrophotometer (Shimadzu UV-160 A, Kyoto, Japan). Glucose solutions with concentrations ranging from 0.02 and 0.1 g/L were used for calibration. A dose–response linear regression was generated using the glucose standard absorbance and results were expressed as g glucose/L.

### 2.6. Total Polyphenols

Total polyphenols were measured colorimetrically by the Folin–Ciocalteau method [28]. Briefly, 0.2 mL of each sample was mixed with 1 mL of Folin–Ciocalteau reagent (diluted 10 times with distilled water). After 5 min, 2.5 mL of sodium carbonate solution (7.5%) was added and the mixture was allowed to stand for 30 min. The absorbance of the resulting solution was measured at 765 nm using a UV–visible spectrophotometer (Shimadzu UV-160A, Kyoto, Japan). Gallic acid was used as a calibration standard and results were expressed as mg gallic acid equivalent (GAE) per liter of sample (mg GAE/L).

### 2.7. In Vitro Total Antioxidant Activity

The total antioxidant activity (TAA) was assessed via the 2,2-azino-bis (ethylbenzothiazoline-6-sulfonic acid) (ABTS) assay by monitoring the reduction of the radical cation as the percentage inhibition of absorbance at 734 nm [29].

The ABTS radical cation was produced by reaction of 10 mL of ABTS stock solution with 100 mL of 70 mM potassium persulfate (K_2_S_2_O_8_) (ABTS:K_2_S_2_O_8_ ¼ 1:0.35 M ratio) and allowing the mixture to stand in the dark at room temperature for 12–14 h before use. After addition of 10 mL of sample (antioxidant) to 10 mL of ABTS work solution, the absorbance at 734 nm was measured every min for a total of 6 min. Results of TAA were expressed in terms of mM of 6-hydroxy-2,5,7,8-tetramethylchroman-2-carboxylic acid (Trolox) equivalent.

### 2.8. Statistical Analysis

Analyses of physicochemical parameters were performed in triplicate. Results were given as mean ± standard deviation. One-way analysis of variance (ANOVA) was used to compare the means. Differences were considered to be significant at *p* < 0.05. Statistical analyses were performed with use of Microsoft Excel software (version 2010; Microsoft Corporation; Redmond, WA, USA).

## 3. Results and Discussion

### 3.1. Optimization Extraction of Antioxidant Compounds from Goji Leaves

A preliminary screening was performed in order to determine the optimal parameters for extraction of antioxidant compounds from Goji leaves. In particular, the purpose of this screening was to identify the optimal extraction conditions in terms of temperature, L/S ratio, and pH, which exert significant influence on the yield of target compounds.

#### 3.1.1. Effect of Temperature on Total Polyphenols and TSS Yields

**Figure 2:**
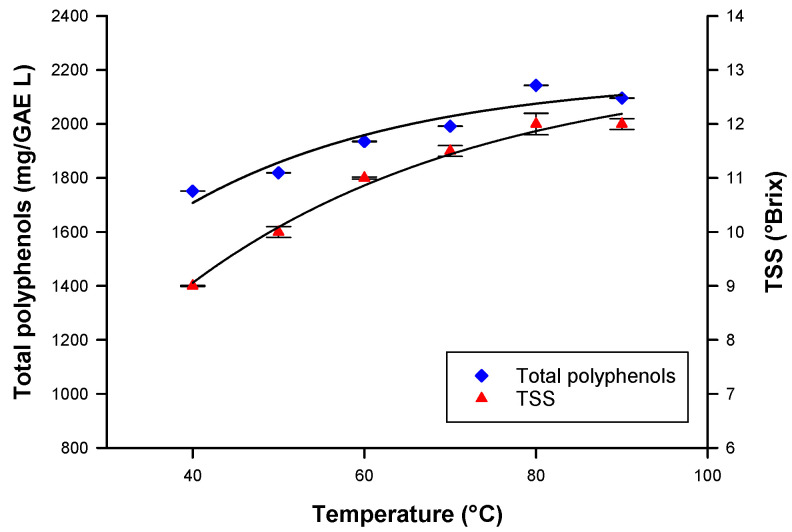
Effect of extraction temperature on total polyphenol and total soluble solid (TSS) yields.

It is well known that the temperature of the extraction solvent is considered one of the most important factors affecting the yields of polyphenols and TSS [30]. The effect of extraction temperature on total polyphenols and TSS yields is reported in Figure 2. Experiments were performed at an operating time of 30 min and an L/S ratio of 4 mL/g. As expected, the total polyphenols and TSS increased gradually with increasing temperature from 40 to 80 °C; a further increase in temperature until 90 °C showed a decrease of polyphenol content, while the TSS content remained constant.

The increase of solvent temperature increased the solubility and diffusion coefficient of solutes, like polyphenols and sugars, decreased both the viscosity of the extracting solvent and surface tension, and consequently enhanced the wetting of leaf particles, resulting in an improved and more efficient extraction process [31,32]. However, the use of temperatures higher than 80 °C decreased the polyphenol yield due to their degradation caused by hydrolysis, internal redox reactions, and polymerization [33,34]. The maximum concentration of soluble matter in the liquid phase was reached at 80 °C. Therefore, 80 °C was selected as the optimal extraction temperature. Similar results were reported by Vuong et al. [35] who studied the effect of extraction temperature (from 5 to 90 °C) on catechin yield from green tea. Results indicated that to obtain a solution enriched in epigallocatechin gallate, the tea should be extracted at 80 °C; temperatures exceeding 80 °C had a noticeable effect on the stability of the epistructured catechins (i.e., epicatechin, epicatechin gallate, and epigallocatechin). An extraction temperature of 80 °C was also selected by Kumar et al. [36] and Bucić-Kojić et al. [37] in the selective extraction of polyphenols from green tea leaves and grape seeds, respectively.

#### 3.1.2. Effect of L/S Ratio on Total Polyphenol, TAA, and TSS Yields

**Figure 3:**
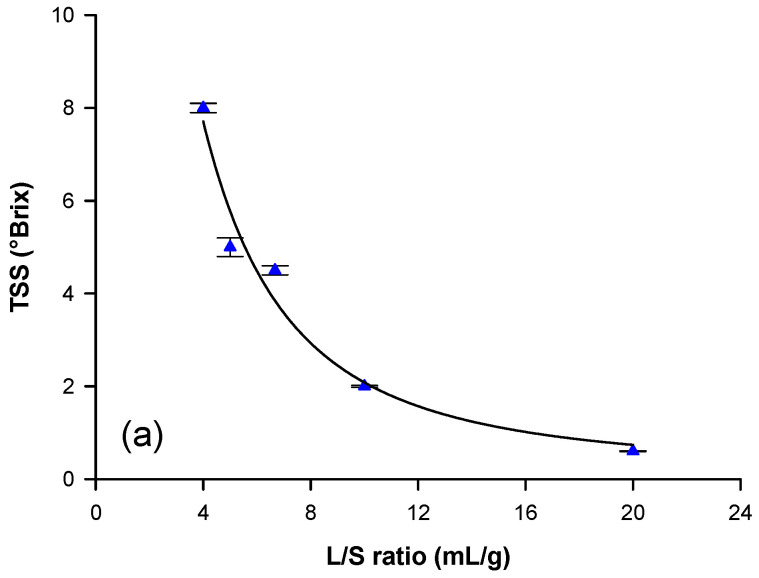

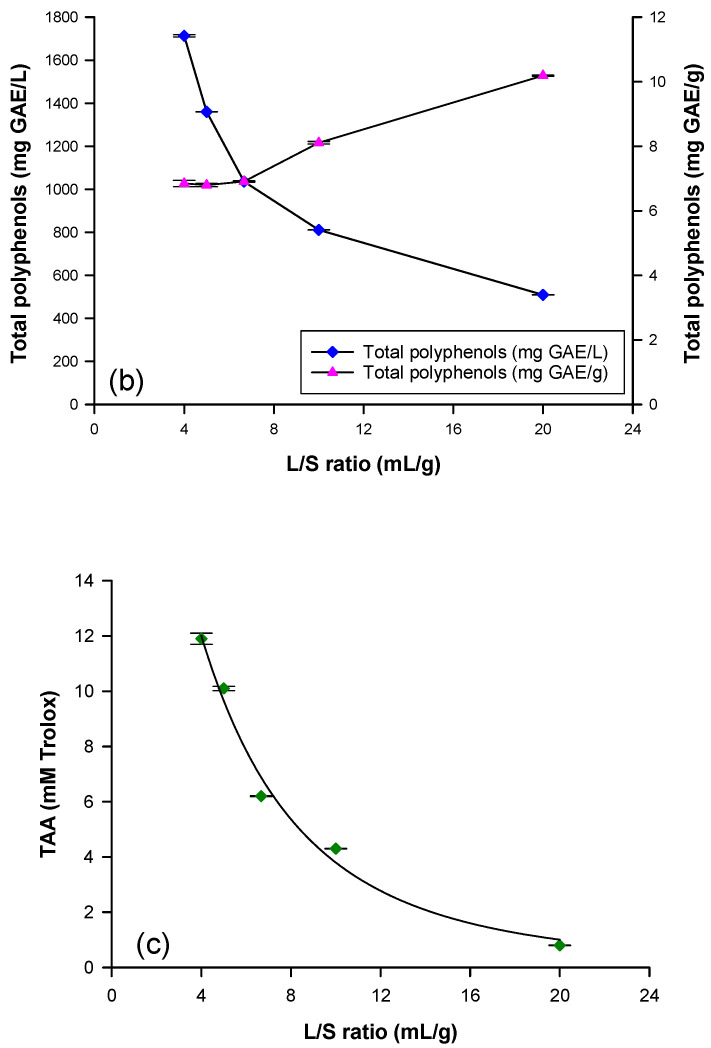
Effect of L/S ratio on (**a**) total soluble solid (TSS), (**b**) total polyphenol, and (**c**) total antioxidant activity (TAA) yields.

The impact of L/S ratio on the extraction of total polyphenols, TAA, and TSS was analyzed and optimized since it affects the yield of specific compounds and, as solvent consumption, exerts a direct influence on the extraction process cost. In particular, the effect of L/S ratio on the yield of total polyphenols, TAA, and TSS was investigated in the range of 4 to 20 mL/g, at the optimal temperature of 80 °C and operating time of 30 min. Figure 3 shows that the concentration of soluble solids and polyphenols, as well as of antioxidant activity, decreased sharply when decreasing the L/S ratio from 20 to 4 mL/g: this means that the extracted compounds were diluted if a higher volume of solvent is used. However, for total polyphenols, the extraction yield, expressed as mg GAE/g, increased with increasing L/S ratio (Figure 3b). Therefore, from an economic point of view, a compromise has to be reached between a high extraction yield and a high polyphenol concentration in the extract. The best condition that fulfilled this requirement was the L/S ratio value in which the curves of extraction yield and concentration of total phenols crossed each other [38], which corresponded to an L/S ratio of 6.6. However, in order to reduce the volume of water needed for the extraction, an L/S ratio of 4 was selected. From an economic perspective, our optimization led not only to the reduction of solvent volume, but also to lower energy consumption, decreasing the overall cost of the process.

A decrease of total polyphenol concentration (mg/L) by increasing the water-to-solid ratio from 8 to 100 mL/g, and an increasing of yield of polyphenols (mg/g) was also observed by Bindes et al. [39], who investigated the effect of L/S ratio on the extraction of polyphenols from green tea leaves. Similarly, Pinelo et al. [40] reported an increase in polyphenol concentration and antioxidant activity of the extract from grape pomace when lower solvent-to-solid ratios were employed. In agreement with present authors, with a reduced amount of solvent, a higher concentration of phenolic compounds in the aqueous extract was attained.

#### 3.1.3. Effect of pH on Total Polyphenols and TSS Yields

**Figure 4:**
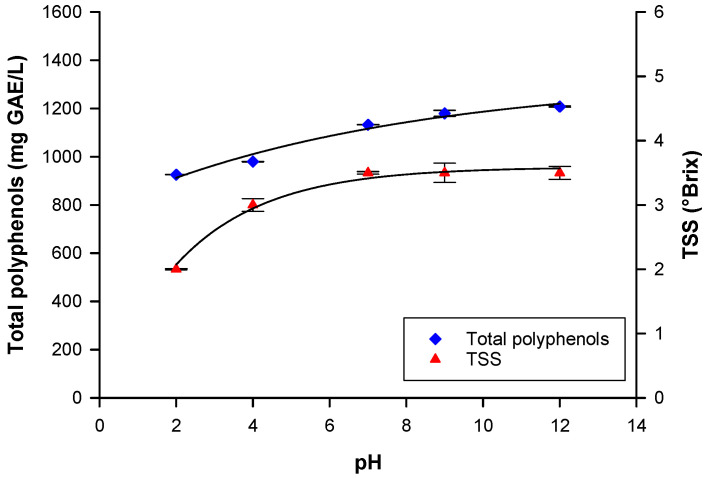
Effect of pH on total polyphenol and total soluble solid (TSS) yields.

The pH of the extraction solution is considered another important parameter that affects the solubility and stability of polyphenols and TSS. The effect of pH on total polyphenol and TSS yields was investigated in the range of 2–12 at an operating temperature of 80 °C. Experimental results showed that at pH values higher than 7 (pH of pristine extraction solution), the yield of total polyphenols in the aqueous extract was slightly higher than at lower pH values (Figure 4). This behavior could be attributed to the fact that mild alkaline hydrolysis breaks ester bonds and releases the ester-bonded phenolic compounds. These results were similar to those reported in other studies. For instance, Boussetta et al. [41] showed increasing levels of polyphenols with increasing NaOH molarity of an extraction solution during the hydroalcoholic extraction of polyphenols from flaxseed hulls by pulsed electric fields. In particular, at a concentration of NaOH of 0.05 mol/L, the polyphenol yields increased up to 3.8 times when compared to an extraction with 0 mol/L. On the other hand, at higher NaOH concentration, a less marked effect was observed.

Rajha et al. [42] reported that the aqueous extraction of polyphenols from vine shoots increased when the pH of extraction solution was increased with NaOH (0.1 M), due to the hydrolysis release of polyphenols linked by ester bonds. However, as shown in Figure 4, a different impact of pH on TSS yield was observed. In particular, with pH values lower than 7, the concentration of TSS was significantly lower; on the other hand, pH values higher than 7 did not produce an increase in TSS. Therefore, considering that the concentration of total polyphenols was not much higher for values greater than 7, the pH value of the pristine extraction solution (pH 7) was selected as the optimum value.

### 3.2. Clarification of Goji Leaf Aqueous Extract

**Figure 5:**
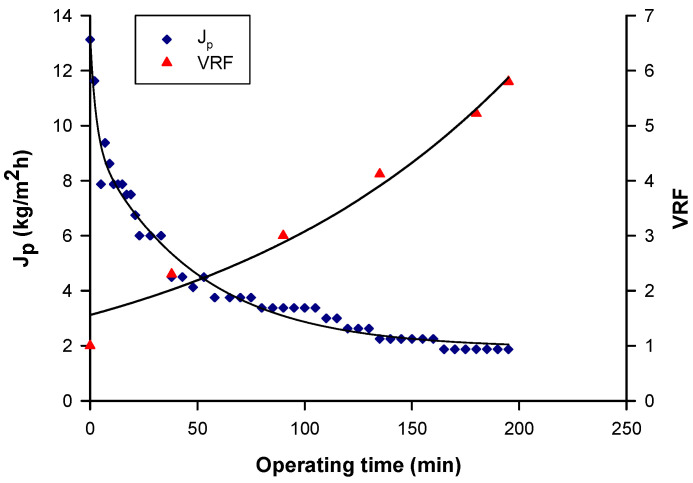
Clarification of goji leaf aqueous extract by ultrafiltration. Time course of permeate flux and VRF (TMP: 0.7 bar; Q_f_: 150 L/h; T: 26 ± 1 °C).

The aqueous extract obtained under the optimized extraction conditions (80 °C, L/S 4 mL/g, 30 min) was clarified by UF in selected operating conditions. Figure 5 shows the time evolution of permeate flux and VRF in the clarification of the aqueous extract with the UF membrane. The initial permeate flux of about 13 kg/m^2^h decreased gradually in the first minute, followed by a gradual decline until a steady-state value of about 2 kg/m^2^h was reached, corresponding to a VRF of 6. The decline of permeate flux can be attributed to the concentration polarization, fouling phenomena, and increased concentration of solutes in the retentate. With increasing VRF, the concentration polarization became more severe. More solutes were convected towards the membrane surface, resulting in a thicker cake layer. This increased the resistance against the solvent flux and the permeate flux declined [43]. A similar behavior was reported in the clarification of vegetable solutions and fruit juices by UF membranes [44,45].

The general composition of aqueous extracts before and after the clarification process is reported in Table 2. The UF treatment allowed the removal of all suspended solids with the production of a clear solution, while the pH remained unchanged in comparison to the unclarified extract. The aqueous extract was characterized by a total soluble solid (TSS) content of 10.5 ± 0.2 °Brix; this value appeared to be higher in comparison with the clarified extract (9.5 ± 0.12 °Brix). This phenomenon can be attributed to the presence of suspended solids in the raw extract, which can interfere with the measurement of the refractive index. The decrease in the content of the total carbohydrates (of about 15%) in the UF permeate could be also attributed to the interaction of carbohydrates with suspended solids.

As previously reported, polyphenols from Goji leaves have attracted great interest in recent years for their health properties [4]. These compounds show significant antioxidant properties largely attributed to their chemical structure. The aromatic features and highly conjugated system with multiple hydroxyl groups make these compounds able to adsorb and neutralize free radicals and other reactive oxygen species (ROS) [46]. Recently, Liu et al. [47] reported that the antioxidant activity of Goji leaves and steams (from three inbred varieties of *Lycium chinense* Miller) was significantly correlated with flavonoid and phenolic contents.

The aqueous extract was characterized by a total antioxidant activity, according to the ABTS method, of 13.0 ± 1.2 mM Trolox, while the content of polyphenols was 1870.7 ± 4.5 mg/L; this value was higher than those reported by Mocan et al. [9] in ethanolic leaf extracts. Both parameters were well preserved in the clarified extract (the rejection of the UF membrane for phenolic compounds and TAA was of 18% and 15%, respectively).

### 3.3. Treatment of Clarified Extract with UF and NF Membranes: Effect of TMP on Permeate Flux and Selectivity

The purification of polyphenols in a complex matrix such as Goji leaf extract is a difficult task, because the membrane performance depends on membrane characteristics, in particular membrane material and pore size or MWCO, as well as on operating conditions. These parameters are closely correlated with fouling mechanisms produced at the membrane surface and inside membrane pores affecting membrane productivity and selectivity towards target compounds [43,48].

In this regard, experiments according to the total recycling configuration were performed at different TMP values in order to evaluate the effect of TMP on both productivity and selectivity towards target compounds for the selected membranes.

**Figure 6:**
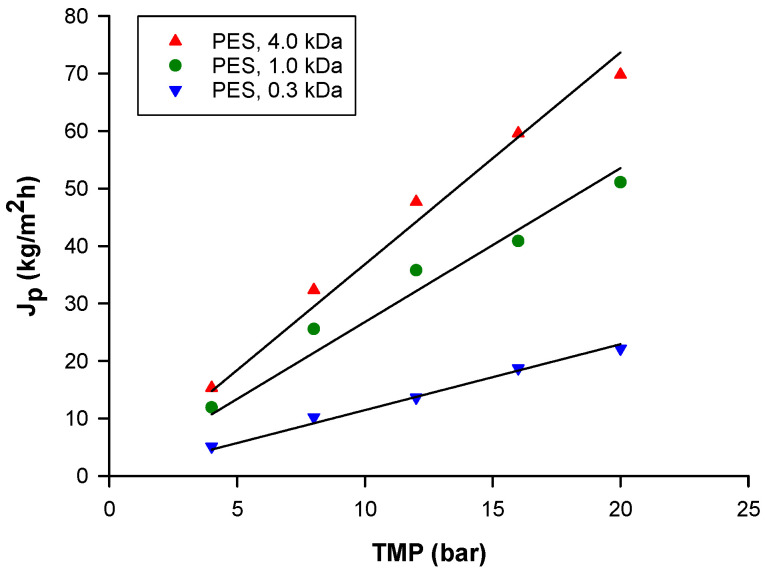
Effect of TMP on steady-state permeate flux for selected membranes (T: 26 ± 1 °C, Q_f_: 580 L/h).

For all the selected membranes, the steady-state permeate flux increased linearly with increasing operating pressure in the range of 4–20 bar (Figure 6). The absence of a limiting flux could be attributed to the preliminary UF treatment, which reduced membrane fouling through the removal of suspended solids. A similar trend was also observed in the concentrations of anthocyanins and phenolic compounds from roselle extract and ultrafiltered bergamot juice, respectively, with NP010 and NP030 membranes [49,50]. As reported in Figure 6, the permeate flux of selected membranes was significantly correlated to their MWCO. In particular, the 4 kDa membrane, with the highest MWCO, showed a higher permeate flux in comparison with the other NF membranes. As expected, this behavior can be attributed to an increasing resistance to the permeate flux exerted by membranes with lower MWCOs [51].

The selected membranes were also compared in terms of fouling index (FI) and cleaning efficiency (CE) (Table 3). The UF membrane (4 kDa) showed the highest FI (54%) and the lowest CE (63%); on the other hand, the 1 kDa membrane showed the lowest FI (22.5%) and the highest CE (100%) after the enzymatic cleaning. These results indicated that internal fouling effects were more effective than cake layer effects during UF with the 4 kDa membrane. On the other hand, the 1 kDa membrane showed the best anti-fouling and cleaning efficiency performance. In particular, the worse FI and CE observed for the 0.3 kDa membrane in comparison to the 1 kDa membrane could be attributed to its higher roughness and contact angle, which in turn led to greater adsorption phenomena [52].

**Figure 7:**
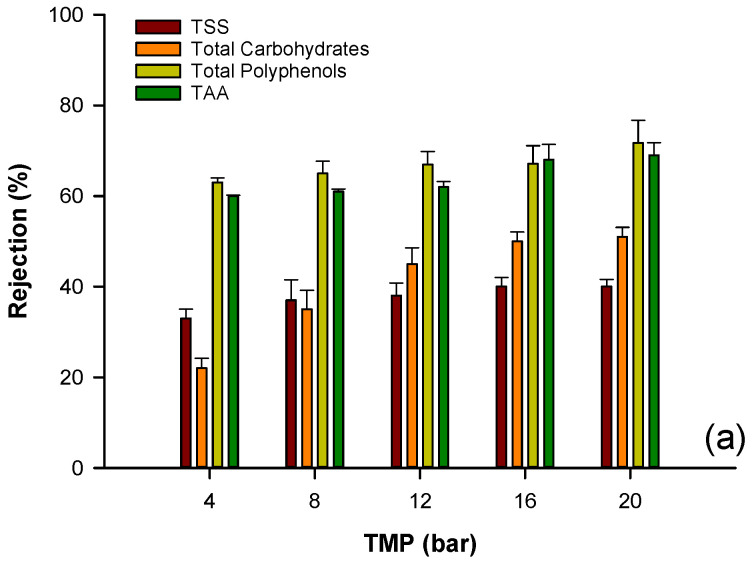

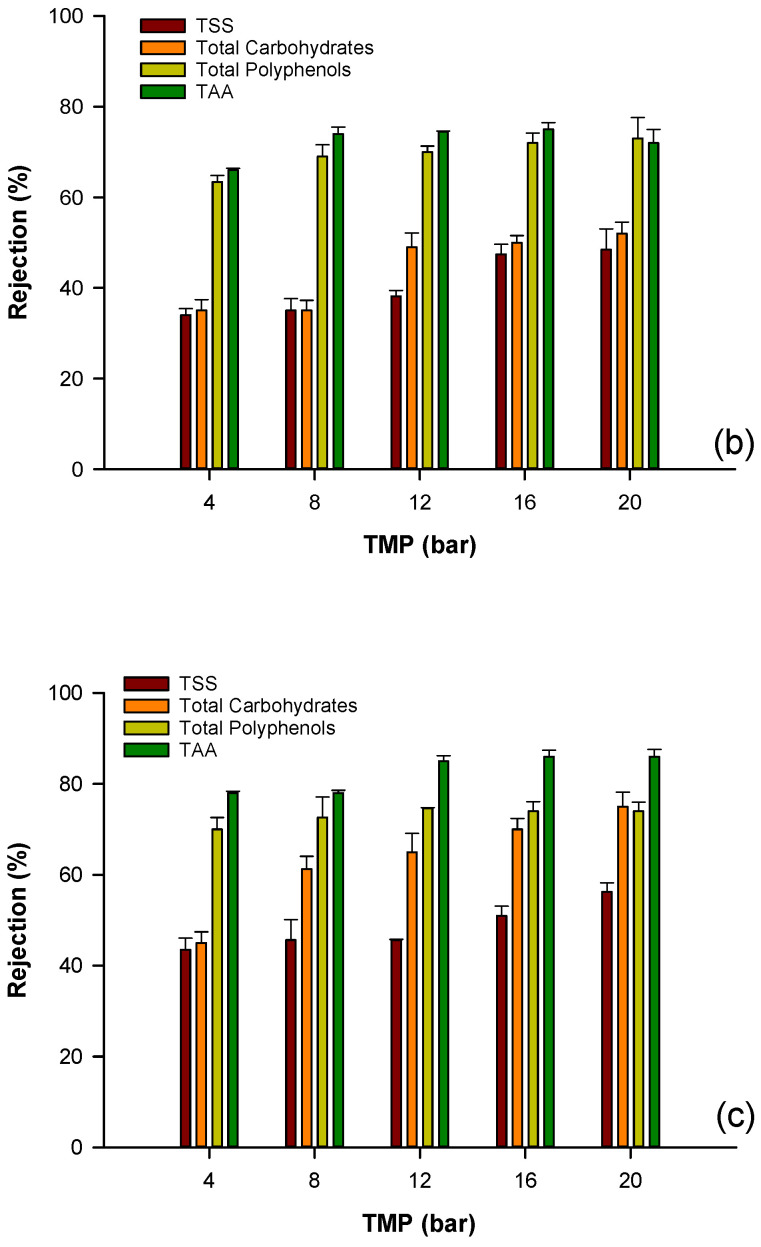
Rejection by UF and NF membranes of analyzed compounds: (**a**) PES 4 kDa; (**b**) PES 1 kDa membrane; (**c**) PES 0.3 kDa membrane.

For all the selected membranes, the rejection of specific compounds was increased by increasing the operating pressure (Figure 7). Specifically, the retention of total carbohydrates and TSS was in the range of 22–51%, 34–52%, and 43–75% for the 4 kDa, 1 kDa, and 0.3 kDa membranes, respectively. A similar trend was also observed for total polyphenols. In this case, the retentions were in the range of 60–71.45%, 63–73%, and 70–86% for the 4 kDa, 1 kDa, and 0.3 kDa membranes, respectively. The obtained results were in agreement with those of several studies that also reported an increase of polyphenol retention with increasing TMP [49,53,54]. This behavior could be attributed to the increased concentration polarization and fouling phenomena, with the formation of a cake layer on the membrane surface and increasing rejection coefficients.

**Figure 8:**
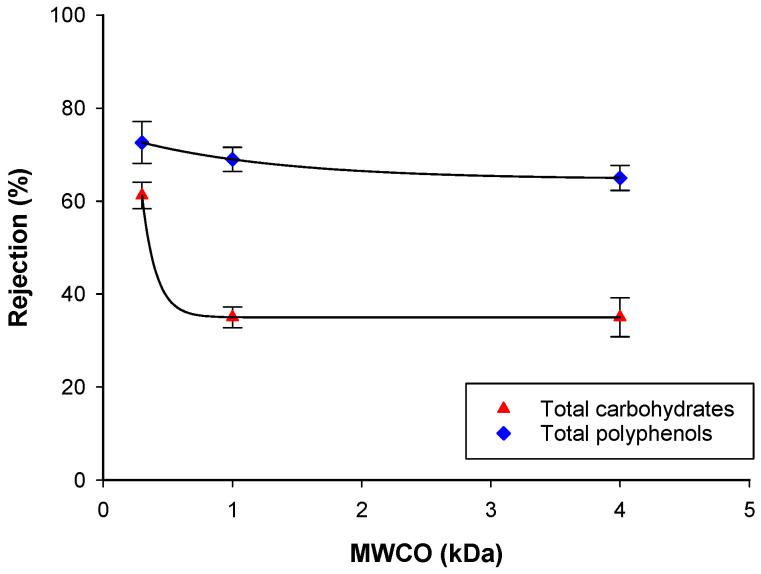
Effect of the MWCO on the rejection by UF and NF membranes of total carbohydrates and total polyphenols (TMP: 8 bar; Q_f_: 580 L/h, T: 26 ± 1 °C).

As shown in Figure 7, the 0.3 kDa membrane showed, at each investigated pressure, the highest rejection of TSS, total carbohydrates, polyphenols, and TAA, in agreement with the lowest MWCO. Consequently, despite the higher value of the rejection of polyphenols and carbohydrates, this membrane was not suitable for the purification of polyphenols and antioxidant compounds. However, a different behavior was observed for the 4 kDa and 1 kDa membranes. In particular, at low pressure values, these membranes showed an improved separation of polyphenols from total carbohydrates. For the 1 kDa membrane, at the operating pressure of 8 bar, the rejection of total carbohydrates was about 35%, while for total polyphenols it was about 70%, indicating for this membrane the best performance in terms of purification of polyphenols (Figure 8) from the clarified aqueous extract. A similar behavior was also observed in previous studies on the recovery of phenolic compounds from bergamot juice and orange press liquor. At a TMP of 6 bar, the 1 kDa membrane showed the largest gap between the rejection coefficients towards sugar and phenolic compounds in the treatment of bergamot juice [50]. At the same TMP value, this membrane showed the lowest average rejection towards sugar compounds (22.8%), and high rejections towards anthocyanins (89.2%) and flavonoids (70%) in the treatment of orange press liquor [55].

It is noteworthy that the measured rejections cannot be explained on the basis of steric considerations alone; other phenomena should be taken into account, including interactions between solutes and the membrane material and association of the solutes with retained macromolecules. It is well known that polyphenols may interact together and with other compounds to form large particles which have a strong contribution to adsorptive fouling. As amphipathic molecules, with hydrophobic aromatic rings and hydrophilic (acidic) phenolic hydroxyl groups, the adsorption of polyphenols onto PES membranes involves both hydrophobic effects and polar interactions (van der Waals, electron donor–acceptor). The formation of multiple hydrogen bonds between polyphenols and the additive polyvinylpyrrolidone (PVP) in PES membranes is considered an additional contributor to adsorption phenomena [48].

### 3.4. Concentration of Clarified Extract with 1 kDa Membrane

On the basis of preliminary results in terms of membrane selectivity and membrane fouling, the 1 kDa membrane was selected for the experiments in batch concentration configuration.

**Figure 9:**
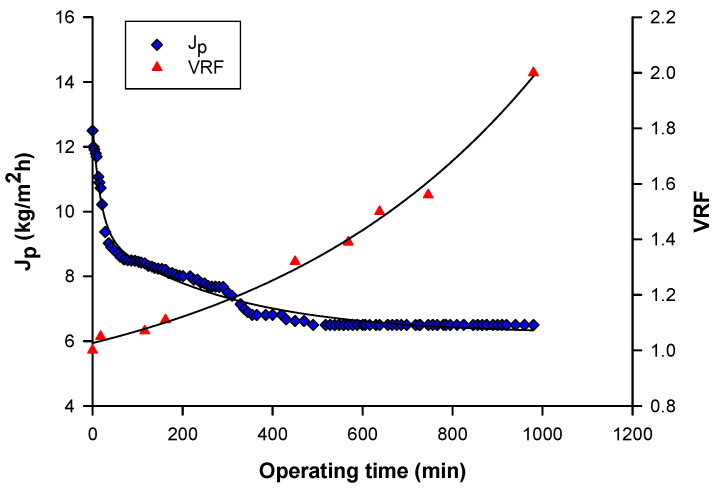
Treatment of clarified aqueous extract with 1 kDa membrane. Time course of permeate flux and VRF (TMP: 8 bar; Q_f_: 585 L/h; T: 26 ± 1 °C).

Figure 9 shows the time evolution of permeate flux and VRF at an applied TMP of 8 bar, an axial feed flowrate of 585 L/h, and temperature of 26 ± 1 °C. In these conditions, the initial permeate flux of 12 kg/m^2^h was reduced up to 6.5 kg/m^2^h when the final VRF of 2 was reached. The analyses of permeate and retentate fractions collected at different VRF revealed that the concentration of polyphenols in the retentate increased along with increasing VRF of the process (Table 4). A similar behavior was obtained in the concentration of phenolic compounds from an aqueous mate (*Ilex paraguariensis* A. St. Hil) extract [56] and aqueous extracts of artichoke [57] by NF membranes. As expected, an increase of the TAA with increasing VRF was also observed. On the other hand, the values of TSS and total carbohydrates remained almost constant in the retentate fractions, and similar to those in the clarified extract.

**Figure 10:**
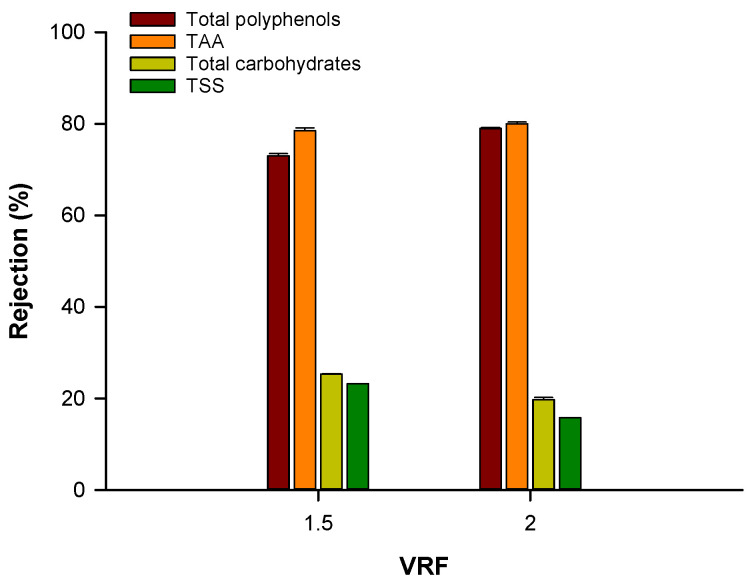
Rejection by the 1 kDa membrane of analyzed compounds as function of the VRF.

These results can be attributed to the lower rejection by the 1 kDa membrane of these components. In particular, as reported in Figure 10, the rejection by this membrane of TSS and total carbohydrates was in the range of 15.8–25.3% and decreased with increasing VRF; accordingly, these compounds were recovered in the permeate fraction. In contrast, the retention values for total polyphenols and TAA were in the range of 73–80% and increased with increasing VRF.

According to the whole results, the 1 kDa membrane allowed the recovery of phenolic compounds in the retentate fraction to be maximized. These fractions may act as antioxidant food additives to provide protection against oxidation and as food supplements, endowing new and relevant properties to foodstuffs and beverages as an alternative to the use of synthetic antioxidants.

## 4. Conclusions

A novel approach based on a combination of aqueous extraction and membrane-based operations was investigated for a more efficient extraction and recovery of phenolic compounds from Goji leaves. Maximum yields were obtained at an extraction temperature of 80 °C, a liquid-to-solid ratio of 4 mL/g, and a pH of 7. The aqueous extract was clarified by ultrafiltration and then fractionated with polyethersulphone membranes, which had MWCOs in the range of 0.3–4 kDa. Among the selected membranes, the 1 kDa membrane showed the highest retention towards polyphenols (higher than 75%) and the lowest retention towards carbohydrates (lower than 25%) at an optimal operating pressure of 8 bar. Therefore, the NF retentate can be considered a valuable fraction enriched in phenolic compounds of interest for the production of health-promoting formulations.

These results could be helpful in the development of large-scale integrated systems for the production at low temperatures of concentrated fractions of *Lycium barbarum* phenolics without thermal damage before final concentration through vacuum evaporation or spray-drying.

## Figures and Tables

**Table 1:** Characteristics of selected membranes.

Membrane Type	UH004P	NP010	NP030
Membrane material	PES	PES	PES
Nominal MWCO (kDa)	4.0	1.0	0.3
Configuration	Flat-sheet	Flat-sheet	Flat-sheet
Operating temperature (°C)	5–95	5–95	5–95
pH range	0–14	0–14	0–14
Water permeability at 25 °C (L/m^2^h bar)	17.12 ^a^	16.0 ^a^	4.53 ^a^
Contact angle (°)	42 (pH 3.8) ^b^	72 (pH 6) ^c^	88 (pH 6) ^c^
Zeta potential at pH 7 (mV)	−7.2 ^d^	−12 ^c^	−15 ^c^
Roughness (Å)	-	13 ^c^	25 ^c^

PES, polyethersulphone; ^a^ our measurement; ^b^ Esmaeli et al. [24]; ^c^ Boussu et al. [25]; ^d^ Lin et al. [26].

**Table 2:** Chemical composition of aqueous extract before and after the UF process.

Parameters	Feed	Permeate	Retentate
Total suspended solids	5.2 ± 2.3	n.d.	6.2 ± 0.1
pH	7.2 ± 0.1	7.1 ± 0.2	7.2 ± 0.8
Total soluble solids (°Brix)	10.5 ± 0.2	9.5 ± 0.12	12 ± 1.2
Total polyphenols (mg GAE/L)	1870.7 ± 4.5	1530.2 ± 2.8	2570 ± 2.6
TAA (mM Trolox)	13.0 ± 1.2	11.0 ± 2.6	14.0 ± 1.6
Total carbohydrates (g glucose/L)	6.33 ± 1.6	5.43 ± 0.12	9.65 ± 2.8

**Table 3:** Water permeability, fouling index, and cleaning efficiency of UF and NF membranes.

Parameter	Membrane Type		
	PES 4 kDa	PES 1 kDa	PES 0.3 kDa
WP_0_ (L/m^2^h bar)	17.12	16.0	4.53
WP_1_ (L/m^2^h bar)	7.85	12.4	2.53
WP_2_ (L/m^2^h bar)	10.77	16.45	4.45
FI (%)	54.0	22.5	44.0
CE (%)	63.0	100	97.6

**Table 4:** Chemical composition of the aqueous extract before and after the treatment with the 1 kDa membrane.

Sample	VRF	Total Polyphenols (mg GAE/L)	TAA (mM Trolox)	Total Carbohydrates (g Glucose/L)	Total Soluble Solids (°Brix)
Feed		1520.0 ± 1.5	9.5 ± 1.4	5.6 ± 0.1	7.2 ± 0.2
Permeate	1.5	525.6 ± 0.9	2.5 ± 0.2	4.3 ± 0.1	5.6 ± 0.1
Permeate	2	534.1 ± 0.2	3.0 ± 0.7	4.8 ± 0.1	6.1 ± 0.4
Retentate	1.5	1961.7 ± 1.1	11.5 ± 2.2	5.6 ± 0.8	7.5 ± 0.3
Retentate	2	2563.6 ± 0.4	15.3 ± 1.0	5.7 ± 0.2	7.6 ± 0.2
